# Influence of center surgical aortic valve volume on outcomes of transcatheter aortic valve replacement

**DOI:** 10.1016/j.xjon.2022.05.010

**Published:** 2022-05-30

**Authors:** Matthew Gandjian, Arjun Verma, Zachary Tran, Yas Sanaiha, Peter Downey, Richard J. Shemin, Peyman Benharash

**Affiliations:** a Cardiovascular Outcomes Research Laboratory, David Geffen School of Medicine at UCLA, University of California, Los Angeles, Los Angeles, Calif; b Division of Cardiac Surgery, David Geffen School of Medicine at UCLA, University of California, Los Angeles, Los Angeles, Calif

**Keywords:** aortic valve replacement, cardiac surgery, clinical outcomes, transcatheter aortic valve replacement, AKI, acute kidney injury, CVA, cerebrovascular accident, ECI, Elixhauser Comorbidity Index, HVH, high-volume hospital, ICD-9/10, *International Classification of Diseases* 9th and 10th revisions, LASSO, least absolute shrinkage and selection operator, LOS, length of stay, LVH, low-volume hospital, MI, myocardial infarction, NCD, National Coverage Determination, NPtrend, nonparametric test of trends, NRD, Nationwide Readmissions Database, SAVR, surgical aortic valve replacement, TAVR, transcatheter aortic valve replacement

## Abstract

**Objective:**

The utilization of transcatheter aortic valve replacement (TAVR) technology has exceeded that of traditional surgical aortic valve replacement (SAVR). In addition, the role of minimum surgical volume requirements for TAVR centers has recently been disputed. The present work evaluated the association of annual institutional SAVR caseload on outcomes following TAVR.

**Methods:**

The 2012-2018 Nationwide Readmissions Database was queried for elective TAVR hospitalizations. The study cohort was split into early (Era 1: 2012-2015) and late (Era 2: 2016-2018) groups. Based on restricted cubic spline modeling of annual hospital SAVR caseload, institutions were dichotomized into low-volume and high-volume centers. Multivariable regressions were used to determine the influence of high-volume status on in-hospital mortality and perioperative complications following TAVR.

**Results:**

An estimated 181,740 patients underwent TAVR from 2012 to 2018. Nationwide TAVR volume increased from 5893 in 2012 to 49,983 in 2018. After adjustment for relevant patient and hospital factors, high-volume status did not alter odds of TAVR mortality in Era 1 (adjusted odds ratio, 0.94; *P* = .52) but was associated decreased likelihood of mortality in Era 2 (adjusted odds ratio, 0.83; *P* = .047). High-volume status did not influence the risk of perioperative complications during Era 1. However, during Era 2, patients at high-volume centers had significantly lower odds of infectious complications, relative to low-volume hospitals (adjusted odds ratio, 0.78; *P* = .002).

**Conclusions:**

SAVR experience is associated with improved TAVR outcomes in a modern cohort. Our findings suggest the need for continued collaboration between cardiologists and surgeons to maximize patient safety.

## Graphical abstract

**Figure:**
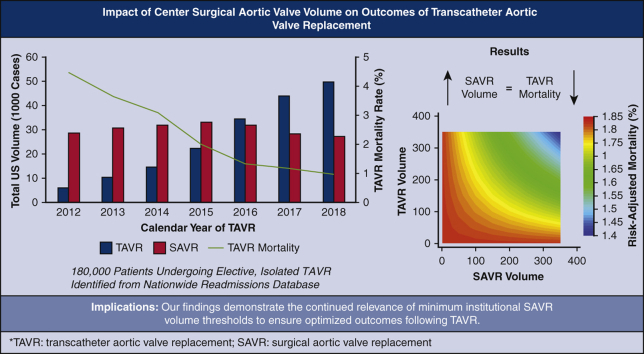

**Figure:**
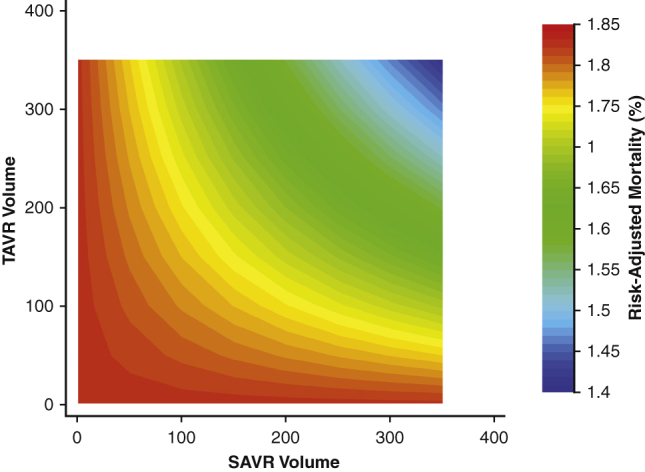
Contour plot of risk-adjusted TAVR mortality by hospital SAVR and TAVR volumes.

**Central Message**

Surgical aortic valve replacement experience is associated with improved transcatheter aortic valve replacement mortality, suggesting the need for continued collaboration between cardiologists and surgeons.

**Perspective**

The Centers for Medicare and Medicare Services national coverage determination includes minimum surgical volume requirements for new TAVR centers. The role of these volume requirements have been recently disputed. A modern analysis regarding the relevance of SAVR volume requirements in ensuring acceptable TAVR outcomes is warranted.

Once reserved solely for patients at prohibitive risk for surgical aortic valve replacement (SAVR), transcatheter aortic valve replacement (TAVR) has emerged as an effective treatment strategy for many with severe, symptomatic aortic stenosis.^1^, ^2^, ^3^, ^4^, ^5^ Recently, new innovations in design coupled with favorable results of randomized clinical trials in low-risk patients have offered increased confidence in this relatively new technology, allowing expanded eligibility for TAVR.^6^, ^7^, ^8^ In fact, the number of Medicare beneficiaries receiving TAVR exceeded SAVR for the first time in the United States in 2017.^9^ Throughout the evolution of TAVR, the concept of shared decision making using a heart team approach has persisted in the United States. Moreover, an advisory panel to the Centers for Medicare & Medicaid Services recommended minimum volume requirements for TAVR, SAVR, and percutaneous coronary interventions to enhance patient safety.^10^ Created in 2012 and updated in 2018, the national coverage determination for TAVR required a minimum of 50 open heart operations during the year before a TAVR center's opening.^11^

Although proponents of minimum volume requirements cite reduced mortality at high-volume SAVR centers, others have noted a more complex interaction, suggesting that SAVR volume alone cannot explain observed TAVR outcomes.^12^^,^^13^ In an important commentary, Barker and colleagues postulated the volume–outcome relationship to be the result of earlier adoption of TAVR and improved patient selection strategies at high-volume centers rather than SAVR experience.^14^ With reduced patient risk and increasing safety profile of TAVR in recent years, a modern analysis regarding the relevance of SAVR volume requirements in ensuring acceptable TAVR outcomes is warranted. In the present study, we sought to evaluate the association of annual hospital SAVR volume on clinical and financial outcomes of TAVR in a contemporary cohort. We hypothesized increasing SAVR caseload to be associated with decreased TAVR mortality and perioperative complications in recent years.

## Methods

### Data Source

This was a retrospective cohort study using data from the 2012-2018 Nationwide Readmissions Database (NRD). Maintained as a part of the Healthcare Costs and Utilization Project, the NRD is the largest, all-payer, nationally representative readmissions database. Robust survey weights are incorporated into all analyses to provide accurate estimates for up to 57.8% of all United States hospitalizations.^15^ The study was deemed exempt from full review by the Institutional Review Board at the University of California, Los Angeles (IRB No. 17-001112, approved July 26, 2017).

### Study Cohort

All elective adult (aged 18 years or older) hospitalizations for TAVR were identified using previously validated *International Classification of Diseases* 9th and 10th revision (ICD-9/10) procedure codes.^13^^,^^16^ Patients with endocarditis as well as those who underwent concomitant open-heart surgery or emergency conversion to sternotomy were excluded to maintain cohort homogeneity (0.4%). Records with missing data for mortality, key demographic characteristics, or hospitalization costs were also excluded (0.6%). Comparison of baseline characteristics between cases with and without missing data is shown in Table E1.

### Variable Definitions

Patient and hospital characteristics, including age, sex, patient income level, and hospital teaching status, were defined in accordance with the NRD data dictionary. The van Walraven modification of the Elixhauser Comorbidity Index (ECI) was used to estimate the burden of chronic conditions.^17^ Briefly, the ECI differentially weighs 31 comorbidities and produces a patient score which ranges from –19 to 89. Additional comorbidities were identified using ICD-9/10 diagnosis codes. Perioperative complications, including cerebrovascular accident (CVA), myocardial infarction (MI), and acute kidney injury (AKI) were similarly ascertained. Using ICD-9/10 diagnosis codes, hemorrhage and accidental puncture were defined as intraoperative events pertaining to any major organ system that complicated the TAVR procedure. Infectious complications were defined as a composite of ICD-9/10 diagnosis codes that have been previously published by our group.^18^

Hospital SAVR and TAVR volumes were independently calculated as the total number of isolated TAVR and isolated SAVR procedures performed at each institution. Because hospitals are not tracked across calendar years in the NRD, operative volumes were generated annually. NRD-provided discharge weights were incorporated into the calculation for volume in congruence with previously reported methodology for absolute volumes.^19^ Hospitalization costs of index admission were obtained by applying center-specific cost-to-charge ratios to overall charges and inflation adjusted to the 2018 Personal Health Index.^20^

The primary outcome of interest was in-hospital mortality following TAVR. Secondary outcomes included CVA, MI, AKI, accidental puncture, hemorrhage, and infectious complications as well as length of stay (LOS), costs, and nonhome discharge.

### Temporal Comparison

To compare the relevance of SAVR volume requirements between a dated and novel cohort of TAVR patients, the study population was split into Era 1 (2012-2015) and Era 2 (2016-2018). This cutoff was chosen to reflect advances in TAVR valve technology and evolving patient candidacy guidelines.

### Designation of High- and Low-Volume Hospitals

Restricted cubic splines were incorporated into a multivariable regression and used to parametrize the relationship between annual hospital SAVR volume and TAVR mortality. Methods used for covariate selection and model development are described below. A 100-bootstrap simulation integrating a Monte Carlo Markov Chain procedure was subsequently performed to identify the SAVR volume, which corresponded to the point of maximum change in risk-adjusted mortality.^21^, ^22^, ^23^ To facilitate analysis of additional TAVR end points, SAVR volume cutoffs were calculated separately in Era 1 and Era 2. Institutions were considered a high-volume hospital (HVH) if they exceeded this threshold or a low-volume hospital (LVH) if their annual SAVR volume fell below the threshold.

### Statistical Analysis

Categorical variables are reported as percentages. Continuous variables are summarized as means with standard deviation (SD) or median with interquartile range, when non-normally distributed. Normality was visually assessed by generating quantile–quantile plots. The χ^2^, adjusted Wald, and Mann-Whitney *U* test were used to compare proportions, means, and medians between groups, respectively. A nonparametric rank-based test by Cuzick was used to assess the statistical significance of nonparametric test of trends (NPtrend).^24^ Multivariable logistic and linear regression models were developed to characterize the association between high SAVR volume status and outcomes of interest, while adjusting for patient comorbidities as well as hospital TAVR caseload. Model covariates were chosen by applying least absolute shrinkage and selection operator (LASSO) methodology.^25^ Briefly, LASSO regularization enhances accuracy and out-of-sample reliability of prediction models by reducing collinearity among selected covariates. LASSO was applied to a training dataset that represented a random 70% sample, whereas model performance was assessed in the remaining 30%. Collinearity between SAVR and TAVR volume was assessed by calculating the variance inflation factor using the *collin* command in Stata. Both volume variables were retained in the final model because the variance inflation factor was less than 10. Covariates selected using LASSO were subsequently included into multivariable regressions and fit using the entire study cohort. Final models were selected based on evaluation of the receiver-operating characteristics (ie, *C* statistic) as well as Akaike and Bayesian information criteria. Regression results are reported as adjusted odds ratios (AOR) or beta coefficients with 95% confidence intervals (CI). All statistical analyses were performed using Stata version 16.1 with α = 0.05 set for significance.

## Results

### Study Cohort and Trends

**Figure 1:**
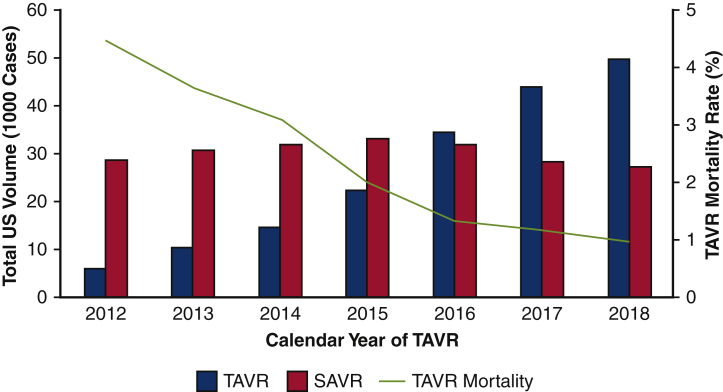
Trends in annual surgical aortic valve replacement (*SAVR*) volume, transcatheter aortic valve replacement (*TAVR*) volume, and TAVR mortality in the United States from 2012 to 2018.

Of an estimated 181,740 patients who met study criteria, 29.3% underwent TAVR during Era 1, and 70.7% during Era 2. Patients in Era 2 were younger (aged 80 vs 81 years; *P* < .001), less commonly female (45.8% vs 47.6%; *P* < .001) and had a lower burden of comorbidities (ECI 5.2 vs 5.3; *P* < .001), compared with Era 1. A comprehensive comparison of additional baseline characteristics and comorbidities between patients in Eras 1 and 2 are shown in Table E2. Over the study period, nationwide TAVR volume increased dramatically, from 5,893 in 2012 to 49,983 in 2018 (NPtrend < .001) (Figure 1). In addition, the number of unique NRD participating hospitals performing elective TAVR rose from 186 in 2012 to 413 in 2018 (NPtrend < .001). Nationwide SAVR volume increased initially from 28,594 cases in 2012 to 33,200 in 2015, followed by a gradual decline in subsequent years culminating with a total of 27,319 in 2018 (Figure 1).

### Hospital Volumes and Comparison of Patients at LVH and HVH in Both Eras

**Figure 2:**
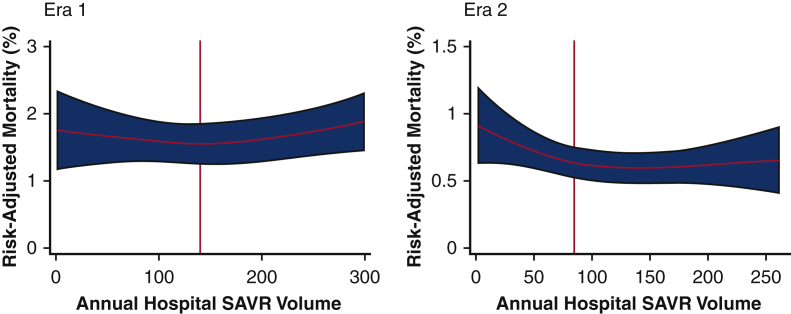
Spline analysis of risk-adjusted transcatheter aortic valve replacement (TAVR) mortality by annual surgical aortic valve replacement (*SAVR*) and TAVR volumes in Era 1 and Era 2.

Based on restricted cubic spline analysis, the optimal threshold associated with decreased TAVR mortality was set at 140 SAVR cases/year in Era 1 and 85 cases/year in Era 2 (Figure 2). In Era 1, HVHs performed 65.3% of TAVR cases. Compared with LVHs in Era 1, patients at HVHs had comparable distributions of age and sex (Table 1). However, HVH patients had a greater median ECI score (5.4 vs 5.2; *P* = .012). Specifically, rates of coagulopathy (18.4% vs 15.4%; *P* < .001) and electrolyte imbalance (21.7% vs 15.4%; *P* < .001) were greater among HVH patients in Era 1, compared with LVH patients. In Era 2, 63.4% of TAVR patients were managed at HVHs. However, in Era 2, patient age, sex, and ECI score were comparable between patients at LVHs and HVHs (Table 1). The incidence of arrhythmia, chronic lung disease, and electrolyte imbalance was higher among HVH patients, relative to LVH patients (Table 1).

**Table 1:** Baseline characteristics stratified by low-volume (LVH) and high-volume (HVH) hospital status

Parameter	Era 1		*P* value	Era 2		*P* value
	LVH (n = 18,456)	HVH (n = 34,777)		LVH (n = 46,998)	HVH (n = 81,509)	
Age (y)	81.1 ± 9.4	81.4 ± 7.5	.19	80.0 ± 8.5	80.0 ± 7.8	.87
Female sex	47.0	47.9	.29	45.9	45.7	.61
ECI score (points)	5.2 ± 1.8	5.4 ± 1.5	.012	5.2 ± 1.7	5.2 ± 1.5	.13
Income percentile			.019			.013
76th-100th	24.6	27.2		23.7	24.6	
51st-75th	26.0	28.2		26.7	28.0	
26th-75th	25.5	26.2		26.2	28.8	
0-25th	23.9	18.4		23.3	18.7	
Payer			.22			.28
Private	6.4	5.2		5.6	5.8	
Medicare	90.9	92.3		91.2	91.6	
Medicaid	0.9	0.7		1.0	0.8	
Other	1.8	1.9		2.3	1.9	
Comorbidities						
Congestive heart failure	31.1	30.7	.87	72.4	72.3	.97
Coronary artery disease	61.2	62.7	.14	62.2	63.3	.13
Chronic arrhythmia	56.2	57.0	.49	51.6	53.0	.017
Pulmonary circulation disorder	16.9	18.6	.10	14.9	15.8	.22
Peripheral vascular disease	22.2	23.4	.28	19.6	19.6	.93
Neurologic disorder	4.9	4.3	.091	4.2	4.3	.52
Chronic lung disease	37.4	38.1	.54	26.3	24.9	.014
Hypothyroidism	16.1	16.9	.21	16.6	17.1	.29
End-stage renal disease	3.6	3.6	.78	4.0	3.8	.22
Liver disease	3.0	2.7	.20	2.3	2.6	.057
Cancer	3.2	3.2	.98	3.1	3.4	.093
Coagulopathy	15.4	18.4	.010	10.3	11.4	.12
Weight loss	3.2	3.0	.51	1.7	2.1	.028
Electrolyte imbalance	15.4	21.7	<.001	9.7	12.2	.035
Anemia	3.2	3.1	.55	3.3	3.4	.35

Values are presented as mean ± SD or %. Comparisons were performed independently among patients in Era 1 (2012-2015) and Era 2 (2016-2018). *ECI*, Elixhauser Comorbidity Index.

### Influence of Volume Designation on In-Hospital Mortality Following TAVR

**Figure 3:**
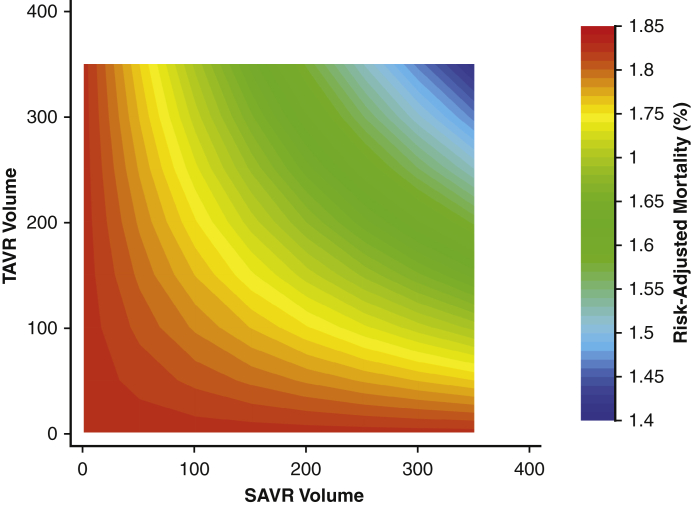
Contour plot of risk-adjusted transcatheter aortic valve replacement (*TAVR*) mortality by hospital surgical aortic valve replacement (*SAVR*) and TAVR volumes.

Over the study period, in-hospital TAVR mortality declined from 4.5% in 2012 to 1.0% in 2018 (NPtrend < .001) (Figure 1). As displayed in Table 2, there was no difference in mortality between HVHs and LVHs in Era 1. However, a subtle, yet statistically significant difference was noted in Era 2 (1.3% vs 1.0%; *P* = .007). After adjustment for relevant patient and hospital characteristics, including TAVR volume, HVH status did not alter the risk of mortality in Era 1 (AOR, 0.94; *P* = .52). Notably, in Era 2, management at HVHs was associated with decreased odds of mortality (AOR, 0.83; *P* = .047). All model coefficients for the multivariable regression to predict in-hospital mortality following TAVR in each Era can be found in Table 3. In addition, a contour plot of risk-adjusted mortality as a function of TAVR and SAVR volume is shown in Figure 3 and demonstrates a gradual decline in mortality associated with increasing TAVR and SAVR volumes.

**Table 2:** Comparison of clinical outcomes and resource use in low-volume (LVH) and high-volume (HVH) hospitals

Outcome	Era 1		*P* value	Era 2		*P* value
	LVH (n = 18,456)	HVH (n = 34,777)		LVH (n = 46,998)	HVH (n = 81,509)	
In-hospital mortality	3.0	2.8	.45	1.3	1.0	.007
Complications						
Accidental puncture	1.6	1.2	.03	0.9	0.8	.47
Acute ischemic stroke	2.1	2.2	.73	0.8	0.8	.99
Acute kidney injury	11.0	10.9	.85	6.2	6.6	.29
Hemorrhage	2.3	2.4	.63	1.6	1.9	.07
Myocardial infarction	1.0	1.0	.68	0.6	0.7	.22
Infectious complication	5.8	6.4	.22	4.3	3.3	<.001
Length of stay (d)	4 (3-7)	5 (3-7)	<.001	2 (1-3)	2 (1-4)	.003
Costs, in $1000s	52.6 (41.2-67.1)	51.3 (41.6-65.1)	.001	44.3 (35.1-56.3)	45.3 (36.1-56.9)	<.001
Nonhome discharge (%)	21.6	25.5	.007	9.1	10.5	.002

values are presented as % or median (interquartile range). Comparisons were separately performed among patients in both Era 1 (2012-2015) and Era 2 (2016-2018).

**Table 3:** Full multivariable logistic regression demonstrating patient and hospital factors associated with in-hospital mortality following transcatheter aortic valve replacement (TAVR)

Parameter	Era 1 (2012-2015)	*P* value	Era 2 (2016-2018)	*P* value
High SAVR volume	0.94 (0.78-1.14)	.52	0.83 (0.69-0.99)	.047
Age, per year	1.02 (1.01-1.04)	.001	1.04 (1.02-1.05)	<.001
Female sex	1.05 (0.90-1.23)	.51	1.33 (1.14-1.56)	<.001
ECI score, per point	0.61 (0.55-0.69)	<.001	0.63 (0.57-0.69)	<.001
Income percentile				
76th-100th	Ref		Ref	
51st-75th	0.96 (0.76-1.21)	.73	1.38 (1.11-1.72)	.004
26th-50th	1.04 (0.83-1.30)	.74	1.03 (0.81-1.32)	.79
0-25th	1.18 (0.94-1.49)	.15	1.42 (1.12-1.81)	.004
Payer status				
Private	Ref		Ref	
Medicare	1.40 (0.88-2.23)	.16	0.89 (0.63-1.24)	.48
Medicaid	0.84 (0.26-2.72)	.78	0.69 (0.30-1.60)	.39
Other payer	1.16 (0.53-2.54)	.71	1.12 (0.60-2.12)	.73
Calendar year				
2012	Ref		–	
2013	0.76 (0.55-1.04)	.089	–	
2014	0.67 (0.49-0.93)	.018	–	
2015	0.48 (0.34-0.68)	<.001	–	
2016	–		Ref	
2017	–		0.98 (0.80-1.21)	.86
2018	–		0.84 (0.68-1.04)	.12
Annual TAVR volume, per case	0.99 (0.99-1.00)	.062	0.99 (0.99-0.99)	.003
Congestive heart failure	1.59 (1.24-2.02)	<.001	2.11 (1.71-2.61)	<.001
Coronary artery disease	0.59 (0.50-0.70)	<.001	0.47 (0.40-0.55)	<.001
Chronic arrhythmia	1.73 (1.39-2.14)	<.001	1.75 (1.46-2.09)	<.001
Pulmonary circulation disorder	1.77 (1.33-2.35)	<.001	1.66 (1.30-2.12)	<.001
Peripheral vascular disease	2.22 (1.73-2.86)	<.001	2.22 (1.80-2.73)	<.001
Neurologic disorder	4.78 (3.61-6.33)	<.001	7.25 (5.86-8.98)	<.001
Chronic lung disease	1.54 (1.19-1.99)	.001	1.10 (0.89-1.36)	.39
Hypothyroidism	0.81 (0.58-1.12)	.21	0.82 (0.61-1.11)	.2
End-stage renal disease	2.85 (1.93-4.20)	<.01	2.35 (1.68-3.29)	<.001
Liver disease	8.75 (8.09-12.6)	<.001	8.26 (5.92-11.5)	<.001
Cancer	1.03 (0.60-1.78)	.92	1.49 (0.92-2.41)	.1
Coagulopathy	1.82 (1.40-236)	<.001	2.19 (1.72-2.78)	<.001
Weight loss	3.61 (2.60-5.00)	<.001	3.85 (2.76-5.37)	<.001
Electrolyte imbalance	3.93 (2.92-5.29)	<.001	8.01 (6.64-9.67)	<.001
Anemia	0.50 (0.24-1.04)	.064	0.47 (0.23-1.00)	.05

Values are presented as adjusted odds ratio (95% CI). *SAVR*, Surgical aortic valve replacement; *ECI*, Elixhauser Comorbidity Index; *Ref*, reference category.

During Era 2, we further examined the relationship between SAVR volume and in-hospital mortality by setting volume cutoffs at 25, 50, and 100 cases per year (2016-2018). We found that 88.4% of centers met the 25 SAVR procedures per year cutoff, 66.3% met the 50 SAVR procedures per year cutoff, and 31.5% met the 100 SAVR procedures per year cutoff. Upon adjusted analysis, only the 100 cases per year SAVR volume cutoff exhibited a statistically significant decrement in TAVR mortality (AOR, 0.80; 95% CI, 0.66-0.97; *P* = .022).

### Influence of HVH Status on Perioperative Complications and Resource Use

Rates of accidental puncture were reduced among patients at HVHs (1.2% vs 1.6%; *P* = .030) during Era 1, but not during Era 2 (0.8% vs 0.9%; *P* = .47). The incidence of CVA, AKI, hemorrhage and MI were similar between LVHs and HVHs durig both Eras, as shown in Table 2. Although rates of infectious complications were equivalent among patients in HVHs and LVHs during Era 1 (6.4% vs 5.8%; *P* = .22), patients at HVHs in Era 2 had lower rates, compared with those at LVHs (3.3% vs 4.3%; *P* < .001). In addition, patients at LVHs experienced longer LOS and accumulated greater hospitalization costs during both Eras, compared with HVH (Table 2). Patients managed at HVHs also faced higher rates of nonhome discharge during both Eras.

Following multivariable regression, HVH status was not associated with altered odds of any perioperative complication during Era 1 (Table 4). However, during Era 2, patients at HVHs had significantly decreased odds of infectious complications, relative to patients at LVHs (AOR, 0.78; *P* = .002). Of note, management at HVHs did not influence patient LOS or total index hospitalization costs during either Era 1 or Era 2 (Table 4). Nonetheless, HVH status was associated with a 25% and 13% increment in relative odds of nonhome discharge during Eras 1 and 2, respectively.

**Table 4:** Risk-adjusted influence of high volume hospital (HVH) status on clinical outcomes and resource use following transcatheter aortic valve replacement (TAVR)

Outcome	Era 1 (2012-2015)	*P* value	Era 2 (2016-2018)	*P* value
In-hospital mortality∗	0.94 (0.78-1.14)	.52	0.83 (0.69-0.99)	.047
Complications∗				
Accidental puncture	0.83 (0.63-1.10)	.20	1.01 (0.80-1.27)	.91
Acute ischemic stroke	1.13 (0.90-1.42)	.28	1.08 (0.81-1.44)	.57
Acute kidney injury	0.89 (0.76-1.04)	.14	1.06 (0.94-1.20)	.35
Hemorrhage	0.98 (0.77-1.24)	.86	0.88 (0.74-1.05)	.17
Infectious complication	1.13 (0.95-1.34)	.18	0.78 (0.66-0.91)	.002
Myocardial infarction	1.04 (0.77-1.40)	.81	1.21 (0.95-1.55)	.12
Hospitalization costs ($1000s)†	+0.2 (-2.7-3.1)	.89	0 (-2.4-2.5)	.97
Length of stay (d)†	-0.1 (-0.3-0.5)	.60	-0.1 (-0.2, 0.1)	.25
Nonhome discharge∗	1.25 (1.06-1.48)	.010	1.13 (1.01-1.27)	.029

Multivariable models included adjustment for all covariates shown in Table 3.

∗ Results of logistic regression tests are presented as adjusted odds ratio (95% CI).

† Results of linear regression tests are presented as β (95% CI).

## Discussion

In the present 7-year-study of more than 180,000 TAVR hospitalizations in the United States, we found rapid adoption of TAVR and a recent decline in utilization of SAVR. Although increasing TAVR volume was associated with reduced odds of mortality during the early years following its market introduction, this effect diminished over time (Video 1). In addition, center-level SAVR volume maintains a strong association with improved mortality following TAVR in more recent years. We found high hospital SAVR volume to be associated with decreased risk of infectious complications but equivalent odds of other inferior outcomes despite a greater burden of patient comorbidities. Several of these findings warrant further discussion and have implications relevant to future updates of National Coverage Determination (NCD).

The rapid adoption of TAVR technology across the United States has been accompanied by major improvements in acute mortality from 4.5% in 2012 to approximately 1% in 2018. Prior studies of the Transcatheter Valve Therapy Registry have demonstrated similar findings using data from a narrower time period.^26^ Although this observation, in part, is due to inclusion of lower-risk patients in recent years, advances in patient selection as well as technical and manufacturing aspects of TAVR technology cannot be ignored. Early TAVR platforms required large diameter delivery sheaths that warranted alternate access strategies, nearly all of which are associated with increased risk compared with the transfemoral approach.^27^^,^^28^ Furthermore, with increasing experience, high-risk features, including dense calcification of the left ventricular outflow tract, have been identified, allowing for better selection of TAVR candidates. Taken together, such advances have allowed for TAVR technology to be adopted into practice for nearly all patients with severe aortic stenosis.

Although a significant positive volume outcome relationship has been demonstrated in many complex surgical and interventional procedures, the presence of this effect in TAVR has been controversial. A study of early TAVR experience demonstrated higher institutional volumes to be associated with significantly lower rate of death as well as hemorrhagic and vascular complications following TAVR.^29^ With aforementioned improvements, some have questioned the relevance of minimum surgical volume requirements set forth by the NCD.^30^ Nonetheless, a study of the Transcatheter Valve Therapy Registry reported a persistent conventional positive outcome relationship even after excluding the learning curve period.^26^ Although our results are congruent with the authors' findings, the present work delves into the potential mechanisms behind the observed relationship. The strong positive association of TAVR mortality and center volume has dissipated in more recent years. This may be due to accumulating operator experience, use of conscious sedation and, of course, lower device-specific risk. With increasing experience, our results suggest continued reductions in mortality and major complications across the United States and the loss of a significant association with annual TAVR volume.

Perhaps the most significant finding of the present study is the association of center-level SAVR volume with TAVR mortality in the moderate risk era. This relationship was present even after accounting for TAVR volume of each center and validates the close interaction of the 2 technologies as initially hypothesized in the original NCD.^10^ Other studies of Transcatheter Valve Therapy Registry have failed to account for such cross-volume effect due to methodologic limitations. Systematic variations across hospitals likely result in collinearity between TAVR and SAVR volumes, with large centers having higher procedural volumes for both and more sophisticated perioperative management.^13^^,^^31^^,^^32^ We especially evaluated the interaction between these modalities and found that emergence of a strong SAVR volume effect on outcomes of TAVR in the latter years of the study. Although the exact reasons for this observation cannot be determined using our dataset, it is possible that hospitals with high surgical expertise in AVR are able to better select patients for TAVR with the heart-team approach. Additionally, the expertise of all members in such a team may help inform the selection of patients who are candidates for alternative surgical intervention, such as video-assisted right minithoracotomy. Our findings provide evidence for the increasing impact of SAVR volume on TAVR-related end points and suggest the need for continued collaboration between cardiologists and surgeons.

The present study is not without limitations. In addition to shortcomings inherent to a retrospective study, the NRD lacks granular clinical information, including echocardiographic findings, ejection fraction, and degree of aortic stenosis. Furthermore, we are unable to ascertain and adjust for the type of valve that was utilized. Because the NRD does not track hospitals across years, we are unable to assess total operative volume across a period >1 year. For the same reasons, the present study was unable to adjust for institutional or surgeon learning curve.

## Conclusions

**Figure 4:**
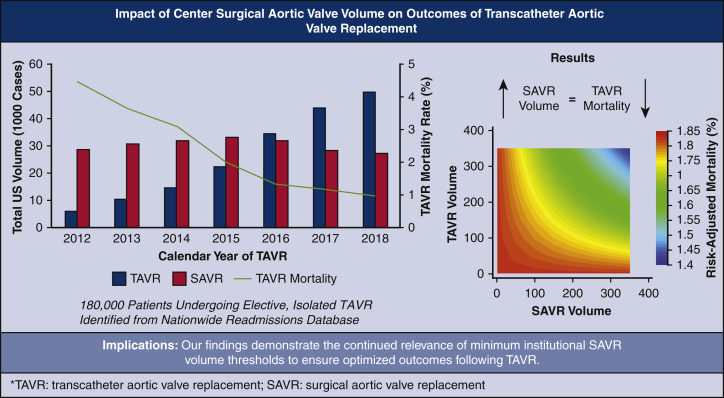
The study's methods, results, and implications. *TAVR*, Transcatheter aortic valve replacement. *SAVR*, surgical aortic valve replacement.

The present study used a nationally representative database to ascertain the influence of institutional SAVR volume on outcomes following TAVR. Using robust statistical techniques to minimize bias in our analyses, we demonstrated the continued relevance of SAVR experience in reducing mortality and perioperative complications in TAVR (Figure 4).

### Conflict of Interest Statement

Dr Shemin served as a consultant to Edwards LifeSciences and as a co-principal investigator on the PARTNER II Trial. All other authors reported no conflicts of interest.

The *Journal* policy requires editors and reviewers to disclose conflicts of interest and to decline handling or reviewing manuscripts for which they may have a conflict of interest. The editors and reviewers of this article have no conflicts of interest.

## Figures and Tables

**Table E1:** Baseline characteristics of study cohort stratified by missingness for key variables, including age, sex, mortality, and hospitalization costs.

Parameter	Not missing (n = 181,740)	Missing (n = 1166)	*P* value
Age (y)	80.4 ± 8.1	80.9 ± 9.6	.21
Female	46.3	47.0	.65
ECI score (points)	5.2 ± 1.6	5.7 ± 1.9	.001
Income percentile			<.001
76th-100th	24.9	55.6	
51st-75th	27.5	27.2	
26th-50th	27.3	9.9	
0-25th	20.4	7.3	
Payer status			.63
Private	5.7	7.3	
Medicare	91.5	92.2	
Medicaid	0.8	0.5	
Other	2.0	0.0	
Comorbidities			
Congestive heart failure	71.4	43.0	<.001
Coronary artery disease	62.7	59.0	.19
Chronic arrhythmia	53.7	56.9	.31
Pulmonary circulation disorder	16.2	14.0	.07
Peripheral vascular disease	20.6	54.1	<.001
Neurologic disorder	4.3	4.3	.91
Chronic lung disease	29.1	28.3	.69
Hypothyroidism	16.8	15.4	.24
End-stage renal disease	3.8	4.8	.002
Liver disease	2.6	5.3	.001
Cancer	3.3	4.0	.14
Coagulopathy	12.8	34.0	<.001
Weight loss	2.3	2.5	.58
Electrolyte imbalance	13.7	13.3	.75
Anemia	3.3	7.9	<.001

Values are presented as mean ± SD or %. *ECI*, Elixhauser Comorbidity Index.

**Table E2:** Comparison of patients in Era 1 (2012-2015) and Era 2 (2016-2018)

Parameter	Era 1 (2012-2015) (n = 53,233)	Era 2 (2016-2018) (n = 128,507)	*P* value
Age (y)	81.2 ± 7.8	80.0 ± 8.2	<.001
Female	47.6	45.8	<.001
ECI score (points)	5.3 ± 1.6	5.2 ± 1.6	.002
Income percentile			.19
76th-100th	26.3	24.3	
51st-75th	27.4	27.5	
26th-50th	26.0	27.8	
0-25th	20.3	20.4	
Payer			.75
Private	5.6	5.7	
Medicare	91.8	91.4	
Medicaid	0.7	0.9	
Other	1.9	2.0	
Comorbidities			
Congestive heart failure	69.1	72.3	.034
Coronary artery disease	62.2	62.9	.31
Chronic arrhythmia	56.7	52.5	<.001
Pulmonary circulation disorder	18.0	15.5	<.001
Peripheral vascular disease	23.0	19.6	<.001
Neurologic disorders	4.5	4.3	.29
Chronic lung disease	37.9	25.4	<.001
Hypothyroidism	16.7	16.9	.46
End-stage renal disease	3.6	3.8	.17
Liver disease	2.8	2.5	.019
Cancer	3.2	3.3	.55
Coagulopathy	17.4	11.0	<.001
Weight loss	3.0	1.9	<.001
Electrolyte imbalance	19.5	11.3	<.001
Anemia	3.1	3.4	.17

Values are presented as mean ± SD or %. *ECI*, Elixhauser Comorbidity Index.

## Supplementary Data

Video 1 Video detailing study methods, results, and implications. Video available at: https://www.jtcvs.org/article/S2666-2736(22)00233-9/fulltext.
