# Supplementary figures and images for: Influence of center surgical aortic valve volume on outcomes of transcatheter aortic valve replacement
Source: JTCVS Open. 2022 May 30;11:62–71. doi: 10.1016/j.xjon.2022.05.010 (PMC9510825; doi:10.1016/j.xjon.2022.05.010)

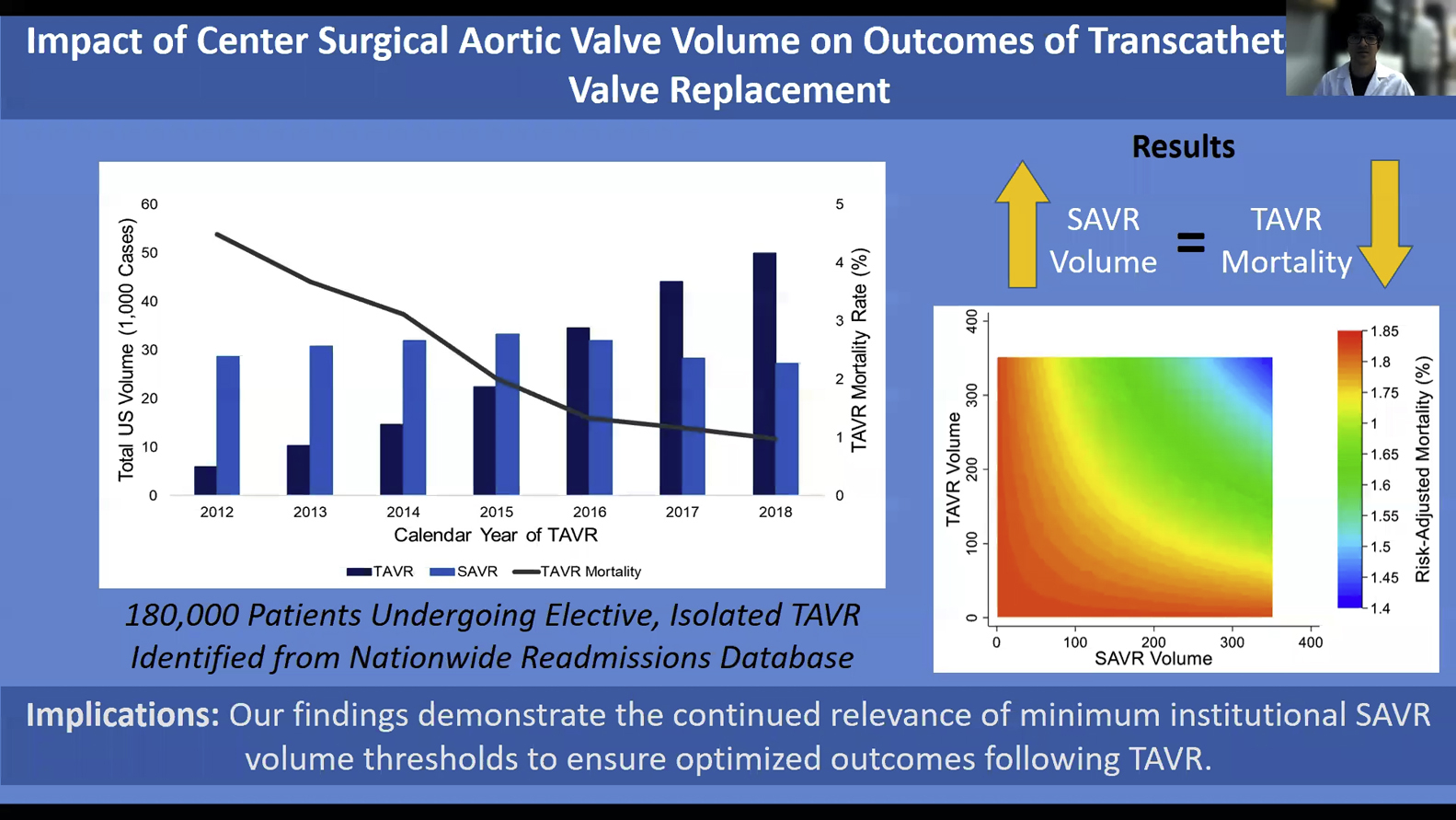

Supplement: Video 1 — Video detailing study methods, results, and implications. Video available at: https://www.jtcvs.org/article/S2666-2736(22)00233-9/fulltext. [file fx3.jpg]
